# Tuberculosis Alleged—Case Cured

**Published:** 1894-09

**Authors:** Edward Cranch

**Affiliations:** Erie, Pa.


### TUBERCULOSIS ALLEGED; CASE CURED.

EDWARD CRANCH, M. D., ERIE, PA.

W. H. S., of Newark, New Jersey, came to his daughter's home in Erie, to be nursed in a sickness that produced rapid exhaustion, with a racking cough and sudden fainting fits, with anorexia as its most prominent features. A specialist was called, who pronounced the case one of tuberculosis, with the tubercle bacilli, and two well-established foci of disease in the lungs.

The color of the border of the tongue was indicative of grippe; the emaciated appearance, as shown by a portrait on the wall, was natural to the patient; the sputa showed no pus, the heart gave a history of strain from overlifting, accounting for the fainting spells.

Sulphur$^{1000}$, one dose, followed later by Kali-bich.$^{1000}$, soon brought up the appetite, and diminished the cough and sputa. A few doses of Rhus-tox.$^{1000}$, and later, of Camphor$^{200}$, banished the fainting spells, and in one month the patient was up and out, going back to work one month later, where he has been every since, in perfect health.

The specialist who treated him had assured him he never would be well, though he might be better. The patient outlived the specialist, who dropped dead in his office, a victim to his own treatment for rheumatic heart disease.

The appearance of the tongue in my case, presenting as it did the characteristic crimson lake border of the grippe, gave me confidence in assuring the man he would recover, and that confidence certainly helped both patient and doctor.

